# 

**Published:** 1890-04

**Authors:** 


### REVISION OF THE UNITED STATES PHARMACOPŒIA.

OFFICIAL ANNOUNCEMENT.

The convention for the revision of the United States Pharmacopœia will be held in the city of Washington, May 7, at noon. As it is necessary that some preliminary arrangements should be prepared in advance of the convention, I have taken upon myself the responsibility of appointing the following delgates to act as a committee of arrangements:

Dr. Samuel C. Busey, Dr. C. H. A. Kleinschmidt, Dr. Robert T. Edes, of Washington; Mr. P. W. Bedford, of New York, and a committee appointed by the National College of Pharmacy of the following members: W. S. Thompson, J. A. Milburn and S. E. Waggaman, M. D.

As soon as arrangements are completed a circular will be mailed to each organization whose credentials are received by me before

April 10, and to any delegate who will forward his address on a stamped envelope inclosed to me.

As president of the convention of 1880, for the revision of the United States Pharmacopœia, I would report that up to the first day of March I have received certificates of credentials from the following list of delegates. If there are any errors in the same, either in initials or names, I would respectfully request that corrections be sent to me, P. O. Box 3281, Boston, Mass. As there are a large number of delegates appointed whose credentials have not yet reached me, a supplementary list will be published later.

[List omitted. The Texas State Medical Association is not mentioned, though credentials were furnished last year.—ED.]

The railroads in the territories of the Trunk Line Commission, Central Traffic Association and the Southern Passenger Association which practically includes all the lines between New York State on the east, Chicago, St. Louis and the Mississippi River on the west, and all the Southern States will make the convention rate of a full fare going, and one-third fare returning (on the usual conditions) to all delegates and their friends to Washington and return. In the New England States and Michigan, as also north of Chicago and west of the Mississippi River no concession can be obtained. Circulars giving fuller particulars will be issued later.

ROBERT AMORY.

[The Texas State Medical Association being a chartered institution is entitled to representation and should send delegates.—ED.]

